# Pd Nanoparticles Immobilized on Pyridinic N-Rich Carbon Nanosheets for Promoting Suzuki Cross-Coupling Reactions

**DOI:** 10.3390/nano14211690

**Published:** 2024-10-22

**Authors:** Shihao Cui, Dejian Xu, Zhiyuan Wang, Libo Wang, Yikun Zhao, Wei Deng, Qingshan Zhao, Mingbo Wu

**Affiliations:** 1 State Key Laboratory of Heavy Oil Processing, College of Chemistry and Chemical Engineering, China University of Petroleum (East China), Qingdao 266580, China; cuishihao001124@163.com (S.C.); xudejian666@163.com (D.X.); 18563407980@163.com (Z.W.); wlb1886056@163.com (L.W.); wumb@upc.edu.cn (M.W.); 2 Qingdao Chaorui Nanotechnologies Co., Ltd., Qingdao 266600, China; zhaoyikun@chaoruinano.com (Y.Z.); dengwei@chaoruinano.com (W.D.)

**Keywords:** Suzuki cross-coupling reaction, pyridinic N-doped carbon, carbon nanosheets, Pd catalysts, metal–support interaction

## Abstract

Palladium (Pd) catalysts play a crucial role in facilitating Suzuki cross-coupling reactions for the synthesis of valuable organic compounds. However, conventional heterogeneous Pd catalysts often encounter challenges such as leaching and deactivation during reactions, leading to reduced catalytic efficiency. In this study, we employed an innovative intercalation templating strategy to prepare two-dimensional carbon nanosheets with high nitrogen doping derived from petroleum asphalt, which were utilized as a versatile support for immobilizing Pd nanoparticles (Pd/N-CNS) in efficient Suzuki cross-coupling reactions. The results indicate that the anchoring effect of high-pyridinic N species on the two-dimensional carbon nanosheets enhances interactions between Pd and the support, effectively improving both the dispersibility and stability of the Pd nanoparticles. Notably, the Pd/N-CNS catalyst achieved an overall turnover frequency (TOF) of 2390 h^−1^ for the Suzuki cross-coupling reaction under mild conditions, representing approximately a nine-fold increase in activity compared to commercial Pd/C catalysts. Furthermore, this catalyst maintained an overall TOF of 2294 h^−1^ even after five reaction cycles, demonstrating excellent stability. Theoretical calculations corroborate these observed enhancements in catalytic performance by attributing them to improved electron transfer from Pd to the support facilitated by abundant pyridinic N species. This work provides valuable insights into feasible strategies for developing efficient catalysts aimed at sustainable production of biaromatic compounds.

## 1. Introduction

Biaromatic compounds serve as important industrial intermediates widely utilized in the production of commercial dyes [1,2], natural products, pharmaceuticals, etc. The Suzuki cross-coupling reaction, which involves the palladium (Pd)-catalyzed cross-coupling of aryl halides with aryl boronic acids, is recognized as one of the most efficient and convenient methods for synthesizing biaromatic compounds [3,4,5]. Conventionally, homogeneous Pd catalysts have demonstrated powerful catalytic capabilities in carbon-carbon coupling reactions [6,7]. However, the sensitivity of Pd-based catalysts to air and the cumbersome recovery process, which causes metal leaching and deactivation, have hindered their broader applications [8]. Therefore, there is an urgent need to develop highly active and stable heterogeneous Pd catalysts as alternatives to their homogeneous counterparts [9,10,11].

Typical support materials, particularly carbon materials such as porous carbon [12,13], activated carbon [14,15], carbon nanotubes [16], graphene [17], etc., have been widely studied and applied in the construction of heterogeneous Pd catalysts. However, the weak interaction between metal nanoparticles and carbon supports often leads to the leaching and deactivation of Pd, resulting in unsatisfactory performance and low metal atom utilization efficiency [18,19]. Consequently, enhancing the metal–support interaction through support engineering has become a prominent research topic [20,21,22]. In particular, nitrogen-doped carbonaceous materials have been identified as a facile and efficacious modification approach [23,24,25], which can diminish the particle size of the metals and foster a homogeneous distribution, thereby augmenting the activity and stability of the catalysts while facilitating Suzuki cross-coupling reactions [26,27,28,29,30]. For instance, Svitlana Pylypenko et al. manifested that nitrogen-doped activated carbon could conspicuously enhance the adsorption of metal particles on the support surface [31]. Søren Kegnaes et al. disclosed that nitrogen-doped carbon-supported metal nanoparticles, encompassing polymeric carbon nitride (PCN) or graphitic carbon nitride (g-C_3_N_4_), demonstrated outstanding heterogeneous catalytic performance [32]. However, the interaction mechanism between the Pd active sites and nitrogen species on the support remains nebulous, which impedes the advancement of advanced Pd catalysts.

In comparison to traditional porous carbon materials [33,34], two-dimensional carbon nanosheets exhibit promising potential as supports for Pd species owing to their open structure for enhanced mass transfer and the availability of efficient nitrogen doping [35,36,37]. Herein, utilizing in situ-formed carbon nitride (g-C_3_N_4_) as the template, a straightforward and effective intercalation templating strategy was proposed for preparing two-dimensional carbon nanosheets with high nitrogen doping derived from petroleum asphalt, which serves as a versatile support for the immobilization of Pd nanoparticles (Pd/N-CNS) for Suzuki cross-coupling reactions. Experimental results, complemented by theoretical calculations, reveal that the pyridinic-rich N species present on the two-dimensional carbon nanosheets effectively improve the dispersibility and stability of the Pd nanoparticles by strengthening the electronic interactions between Pd and the support. Remarkably, the prepared Pd/N-CNS catalyst achieved exceptional catalytic performance and stability towards the Suzuki cross-coupling reactions under mild conditions, achieving an approximately 9-fold increase in activity compared to commercial Pd/C catalysts. This work offers a feasible approach for the development of efficient heterogeneous Pa-based catalysts for the synthesis of valuable organic compounds.

## 2. Materials and Methods

### 2.1. Chemicals and Reagents

Pd_2_(dba)_3_·CHCl_3_ (Pd, 20.4 wt%), toluene (99.0 wt%), palladium acetate (Pd, 46.0–48.0 wt%), dicyandiamide (analytical purity), anhydrous K₂CO₃ (analytical purity), bromobenzene (99.0 wt%), phenylboronic acid (97.0 wt%), acetone (99.0 wt%), and ethanol (99.5 wt%) were acquired from Aladdin Chemical Reagent Co., Ltd. (Shanghai, China). The petroleum asphalt (Grade 70) was sourced from Sinopec Jiujiang Branch (Jiujiang, China).

### 2.2. Preparation of the Samples

#### 2.2.1. Preparation of Nitrogen-Doped Carbon Nanosheets (N-CNS)

In total, 1 g of petroleum asphalt was dissolved in 50 mL of toluene and subjected to stirring and dispersion for 10 min. Subsequently, 4 g of dicyandiamine (DICY) was added to form a uniform suspension. The toluene was removed through vacuum distillation, yielding a solid powder. The powder was transferred to a tubular furnace, where it was heated to 550 °C in a N_2_ atmosphere and calcined for 4 h to produce a block asphalt carbon (BAC) embedded with a carbon nitride (g-C_3_N_4_) template. Thereafter, the BAC material was subjected to calcination for 2 h temperatures ranging from 800 to 1000 °C, with a heating rate of 5 °C min^−1^ in a N_2_ atmosphere, removing the template and yielding N-CNSt samples (t = 800, 900, 1000).

#### 2.2.2. Preparation of Nitrogen-Doped Carbon Nanosheets-Supported Palladium Catalyst (Pd/N-CNS)

In total, 50 mg of N-CNSt sample was dispersed in 20 mL of chloroform, to which 5 mg of Pd_2_(dba)_3_·CHCl_3_ was added and mixed using ultrasonic treatment. The resulting mixture was refluxed at 80 °C for 30 min, after which the solid product was isolated by repeated washing with acetone and water. The Pd/N-CNS catalysts were subsequently obtained through a freeze-drying procedure. For comparative purposes, Pd/AC and Pd/rGO catalysts were prepared by using a similar procedure except for changing N-CNSt to activated carbon (AC) and reduced graphene oxide (rGO). The Pd(II)/N-CNS800 catalysts were prepared by changing Pd_2_(dba)_3_-CHCl_3_ to palladium acetate and by impregnating the palladium acetate onto N-CNS800, with the rest of the process being similar to that described above for the catalysts. Each catalyst sample was prepared three times under the same conditions and the data results were averaged.

### 2.3. Catalytic Performance for Suzuki Cross-Coupling Reactions

In the standard procedure for carbon–carbon coupling reactions, bromobenzene (1 mmol, 159.0 mg), phenylboronic acid (1.5 mmol, 190.0 mg), anhydrous K₂CO₃ (2 mmol, 279.3 mg), and the Pd/N-CNS catalyst (0.05 mmol% Pd) were introduced into a Schlenk tube and thoroughly dispersed in a 10 mL alcohol–water mixture (3:1). Following three rounds of Ar degassing, the mixture was subjected to a 100 °C oil bath for a reaction duration of 50 min. Upon completion of the reaction, the solution was analyzed using gas chromatography (GC). The catalyst was separated from the reaction system and washed with water, rendering it available for subsequent reaction cycles. Data and error bars represent the average and standard deviation of data from triplicate parallel tests.

### 2.4. Characterization

The morphology of the samples was investigated by high-resolution transmission electron microscopy (HR-TEM) (FEI, Tecnai G2 F20, Waltham, MA, USA). X-ray diffraction (XRD) was conducted on an X-ray diffractometer of X’Pert Pro MPD type with a Cu K source (40 kV, 40 mA). The X-ray photoelectron spectroscopy (XPS) analyses were performed on a Thermo Scientific K-Alpha X-ray photoelectron spectrometer. The N_2_ adsorption–desorption isotherms were obtained at 77 K using automatic volumetric adsorption equipment (Belsorp-max, Osaka, Japan). A gas chromatograph (GC) from Beijing Beifen Ruili Analytical Instruments Co., Ltd., (BF-6050, Beijing, China) was utilized to test the reaction product composition and content.

### 2.5. DFT Calculations

The VASP software package (VASP 5.4.1) was used to optimize the structural configuration of the model and calculate the energy and electronic properties. The exchange–correlation energy was solved by generalized gradient approximation (GGA) and the Perdew–Burke–Ernzerhof (PBE) functional [38], and the truncation energy was set to 500 eV. Automatic k-point sampling was implemented with a grid of 5 × 5 × 1, and the convergence of energy and force were set to 1 × 10^−5^ ev and 0.01 V·Å^−1^, respectively. The thickness of the vacuum layer in the Z direction of the model plane is configured to 15 Å to avoid the interaction caused by periodicity.

## 3. Results and Discussion

**Figure 1:**
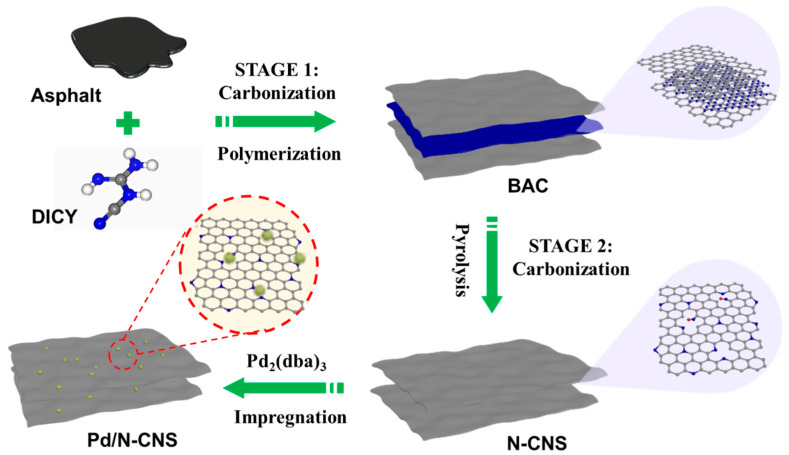
Schematic illustration of the preparation procedure of the Pd/N-CNS catalysts.

Figure 1 illustrates the preparation procedure of the Pd/N-CNS catalyst. Initially, petroleum asphalt was combined with dicyandiamide (DICY) and subjected to an initial calcination process. During this phase, DICY underwent polymerization to form graphitic carbon nitride (g-C_3_N_4_), establishing a templated intercalation architecture that resulted in bulk carbon material (BAC) intercalated with the g-C_3_N_4_ template. The X-ray diffraction (XRD) pattern of BAC reveals characteristic peaks at 13.1° and 27.4°, corresponding to the (100) and (002) diffraction planes of g-C_3_N_4_ (Figure S1a). The scanning electron microscopy (SEM) image confirms the layer-by-layer sandwich-like structure of the BAC intermediate, as depicted in Figure S1b. Subsequently, a second calcination step was performed to remove the template, during which nitrogen was incorporated to yield nitrogen-doped carbon nanosheets (N-CNSs), resulting in two-dimensional carbon nanosheets with high nitrogen content. Significantly, by fine-tuning the secondary calcination temperature during template removal, the structural morphology, nitrogen doping content, and its type can be effectively modulated. An increase in calcination temperature was found to promote a tendency for stacking within the lamellar structure of the resulting N-CNSt materials, as demonstrated by the SEM and transmission electron microscopy (TEM) images presented in Figure S2. Furthermore, high-resolution TEM images reveal that N-CNS800 exhibits a two-dimensional structure with a higher density of surface defects compared to N-CNS900 and N-CNS1000. Finally, Pd/N-CNS catalysts were prepared by depositing Pd nanoparticles onto the NCNSt substrates using a facile impregnation method. The nitrogen doping and abundant defects on the support strengthen the anchoring of palladium nanoparticles, rendering them highly effective for Suzuki cross-coupling reactions.

**Figure 2:**
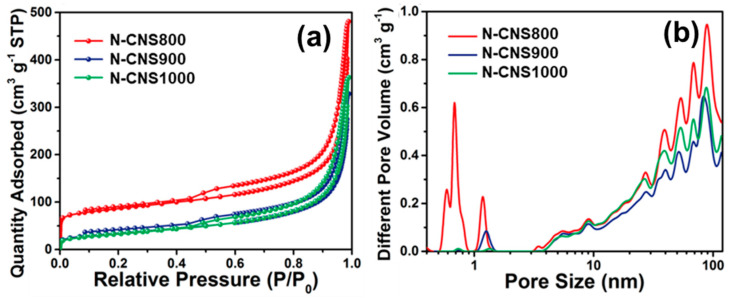
(**a**) N_2_ adsorption–desorption isotherms and (**b**) pore size distribution of N-CNS800 (red), N-CNS900 (blue), and N-CNS1000 (green).

The structural characteristics of the N-CNSt samples were examined via XRD, Raman spectroscopy, and FTIR analysis (Figure S3). The XRD patterns of the N-CNSt samples reveal that the peaks observed at 25.0° and 43.8° correspond to the characteristic reflections of the C (002) and (100) crystal planes, respectively, thereby indicating an amorphous carbon structure. The Raman spectra indicate that the I_D_/I_G_ ratios for the N-CNSt samples are measured at 1.16, 1.11, and 1.05, respectively. This trend suggests a notable reduction in defect density within the samples as the calcination temperature increases, attributed to g-C_3_N_4_ pyrogenic decomposition. Moreover, the emergence of a 2D characteristic peak at 2750 cm^−1^ corroborates that the N-CNSt samples exhibit a graphene-like structure. The FTIR spectrum of BAC exhibits a characteristic peak at approximately 810 cm^−1^, corresponding to the triazine unit structure of g-C_3_N_4_, which is further substantiated by the C-N heterocyclic stretching vibration peaks observed at 1260, 1339, 1420, 1575, and 1630 cm^−1^. After secondary calcination, the FTIR spectra of resulting N-CNSt samples indicate the disappearance of the characteristic peaks of g-C_3_N_4_, confirming the complete decomposition of the g-C_3_N_4_ intermediate and demonstrating that the nitrogen atoms within the material predominantly reside embedded in the carbon framework. To further elucidate the porosity characteristics of the NCNSt materials, nitrogen adsorption–desorption isotherms, and pore size distribution analyses were conducted. All three materials exhibit distinct hysteresis characteristics of type-IV isotherms (Figure 2a), with BET-specific surface areas recorded at 286.8, 124.3, and 121.2 m²·g^−1^, respectively (Table S1). The corresponding pore size distribution (Figure 2b) indicates that N-CNS800 is distinguished by a more abundant microporous structure, aligning with the observations in the TEM analysis.

**Figure 3:**
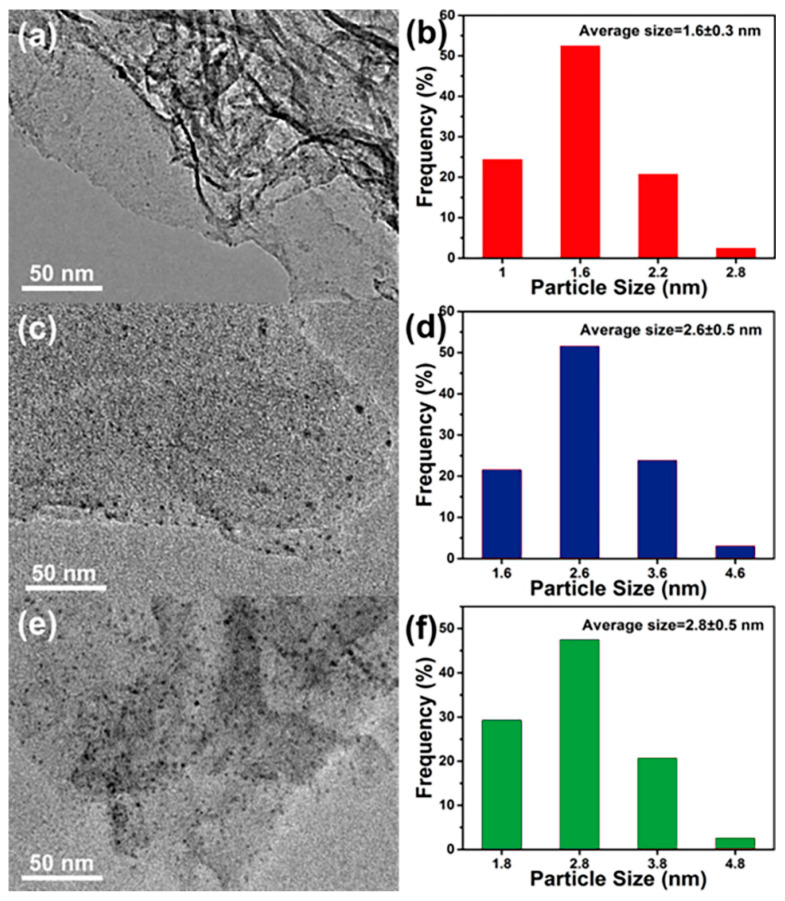
TEM images and corresponding palladium particle size distribution of (**a**,**b**) Pd/N-CNS800 (red), (**c**,**d**) Pd/N-CNS900 (blue), and (**e**,**f**) Pd/N-CNS1000 (green).

TEM characterization was conducted to evaluate the morphology and microstructure of the synthesized Pd/N-CNS catalysts. As illustrated in Figure 3, the Pd/N-CNS800, Pd/N-CNS900, and Pd/N-CNS1000 catalysts exhibit a uniform distribution of palladium nanoparticles dispersed across the two-dimensional substrates, with average particle sizes measuring 1.6 ± 0.3 nm, 2.6 ± 0.5 nm, and 2.8 ± 0.5 nm, respectively. High-resolution transmission electron microscopy (HR-TEM) further confirms the distinct attachment of these particles to the support (Figure S4), revealing a lattice spacing of approximately 0.22 nm corresponding to the (111) facet of palladium. Notably, with a calcination temperature increase, there is a marked increase in the quantity of Pd nanoparticles on the catalyst surface, leading to a broader particle size distribution and an elevation in the average particle size. Considering the structural properties of the support, it becomes evident that the variations in metal nanoparticles on the catalyst surface are profoundly influenced by the nitrogen doping level of the support. The adsorption and stabilization of Pd nanoparticles are particularly favored in N-CNS800, which is characterized by a high nitrogen doping level and surface defects. With increasing calcination temperature, a reduction in stable sites for anchoring Pd nanoparticles occurs due to a decrease in the nitrogen doping level of the support. Consequently, catalysts synthesized at elevated temperatures are marked by insufficient dispersion and uneven distribution of metal nanoparticles on the carbon nanosheet surfaces.

**Figure 4:**
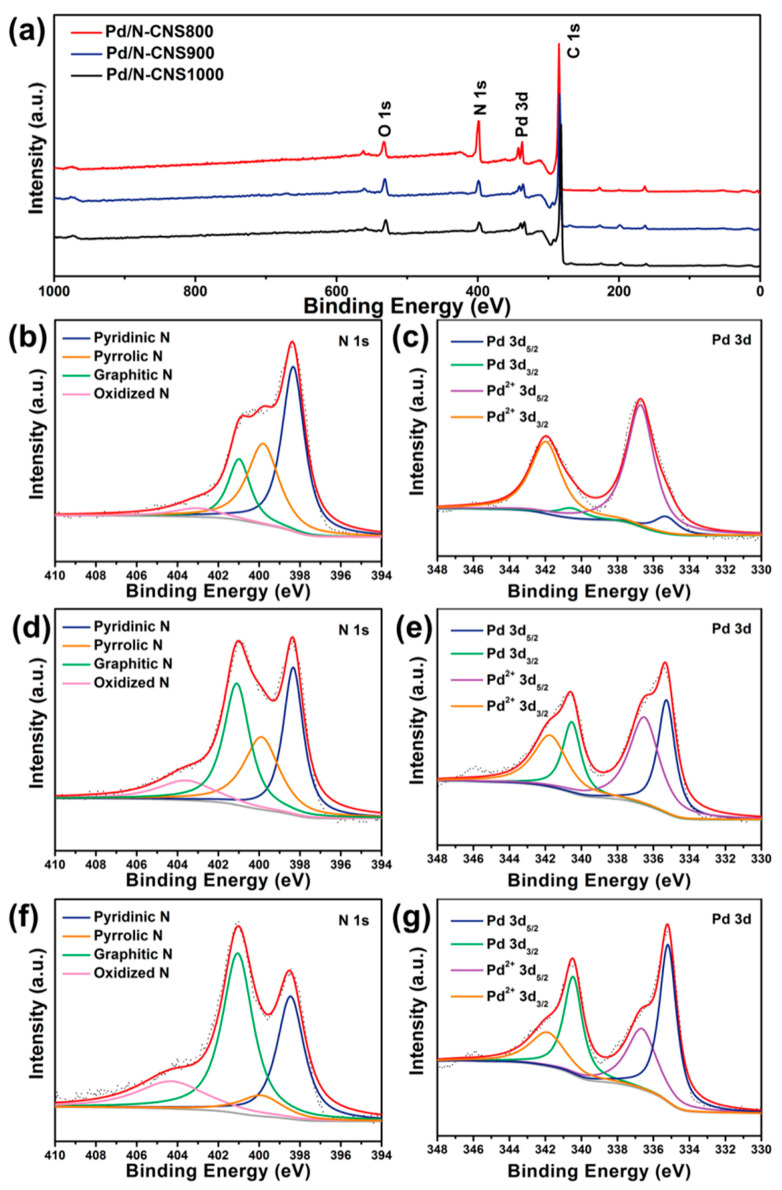
(**a**) Full-range XPS survey spectra of the Pd/N-CNS samples. High-resolution N 1 s and Pd 3d XPS spectra of (**b**,**c**) Pd/N-CNS800, (**d**,**e**) Pd/N-CNS900, and (**f**,**g**) Pd/N-CNS1000.

X-ray photoelectron spectroscopy (XPS) analysis was conducted to uncover the valence bond structure of the Pd/N-CNS catalysts. The full-range XPS spectra reveal the presence of C, O, N, and Pd elements within the samples (Figure 4a). The high-resolution N 1 s XPS spectra of Pd/N-CNS800, Pd/N-CNS900, and Pd/N-CNS1000 are depicted in Figure 4b,d,f, which can be deconvolved into four distinct peaks attributed to pyridinic N, pyrrolic N, graphitic N, and oxidized N, with peak positions centered at 398.5, 401.3, 285.9, and 404.5 eV, respectively. The nitrogen atomic ratios of Pd/N-CNS800, Pd/N-CNS900, and Pd/N-CNS1000 materials are 14.18%, 8.06%, and 5.96%, respectively, indicating a stepwise decrease in nitrogen content with a rise in calcination temperature (Table S2). The high-resolution Pd 3d XPS spectra of the Pd/N-CNS catalysts show that the palladium species are present in both Pd^0^ and Pd^2+^ states, as depicted in Figure 4c,e,g. The binding energies of Pd^0^ 3d_3/2_ and 3d_5/2_ are recorded at 335.3 and 340.5 eV, respectively, while those for Pd^2+^ 3d_3/2_ and 3d_5/2_ are situated at 336.7 and 342.0 eV. According to inductively coupled plasma optical emission spectroscopy (ICP-OES) analysis, the palladium loading in Pd/N-CNS800, Pd/NCNS900, and Pd/NCNS1000 is determined to be 0.69 wt.%, 1.03 wt.%, and 1.24 wt.%, respectively (Table S2).

**Figure 5:**
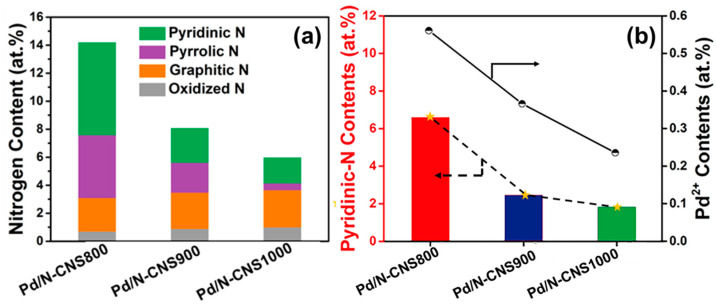
(**a**) Nitrogen content of each type of N species in the Pd/N-CNS samples. (**b**) Pyridinic-N contents of the Pd/N-CNS samples and corresponding Pd^2+^ contents.

The influence of nitrogen doping on the Pd active sites was further investigated. The distribution of various nitrogen species within the Pd/N-CNSt materials is illustrated in Figure 5a. In Pd/N-CNS800, the predominant forms are pyridinic and pyrrolic nitrogen species. As the secondary calcination temperature escalates to 900 and 1000 °C, a substantial reduction in nitrogen content in Pd/N-CNS900 and Pd/N-CNS1000 is observed, primarily attributed to the decomposition of pyridinic and pyrrolic nitrogen moieties due to their lower thermal stability at elevated temperatures. Remarkably, pyridinic nitrogen atoms can form coordinate bonds with palladium atoms, enhancing the stability of nanoparticles by mitigating agglomeration. This type of nitrogen has been demonstrated to effectively interact with palladium surfaces, underscoring its role in preserving nanoparticle dispersion. Pyrrolic nitrogen, on the other hand, enhances the electron density around palladium, which can improve the metal’s resistance to oxidation and aggregation. In comparison, it is observed that the increase in nitrogen content not only results in a significant reduction in metal loading and nanoparticle size but also promotes the conversion of palladium species from Pd^0^ to Pd^2+^. Given that Pd_2_(dba)_3_·CHCl_3_ was selected as the palladium precursor, the elevation of the palladium valence state highlights the pronounced electron-withdrawing effect exerted by the nitrogen-doped carbon support. This observation is further corroborated by XPS analysis of Pd/AC, which was prepared through direct impregnation of Pd_2_(dba)_3_·CHCl_3_ onto activated carbon (AC). It is evident that palladium nanoparticles were primarily adsorbed and grew on the surface of AC without any indication of oxidized palladium states (Figure S5). Notably, Figure 5b depicts a correlation between pyridinic N content and corresponding Pd^2+^ levels within the Pd/N-CNS catalysts. The linear relationship demonstrates that pyridinic N plays a crucial role in facilitating electron interactions between palladium and its support material. Among these catalysts, Pd/N-CNS800 exhibits the highest pyridinic N content at 6.59 at.%, effectively stabilizing deposited palladium species while enhancing their dispersibility, catalytic efficiency, and stability.

To evaluate the catalytic efficacy of the prepared Pd/NCNS catalysts, the Suzuki cross-coupling reaction between bromobenzene and phenylboronic acid was employed as a probe reaction, as illustrated in Table 1. The results indicate that the reaction system remains inert in the absence of a catalyst (entry 1), underscoring the indispensable role of the catalyst. The N-CNSt displays low conversion rates (entries 2–4), which are notably enhanced upon the incorporation of Pd NPs onto the supports, thus affirming the high catalytic efficiency of Pd in the Suzuki cross-coupling reaction (entries 5–7). As calculated, the overall turnover frequency (TOF) values for Pd/N-CNS800, Pd/N-CNS900, and Pd/N-CNS1000 are 2390, 2083, and 2179 h^−1^, respectively, among which Pd/N-CNS800 exhibits the highest overall TOF. Under identical reaction conditions, the yield of Pd(II)/N-CNS800 is only 85.7% after 50 min of reaction, with a relatively lower overall TOF value of 2057 h^−1^ (entry 8). Furthermore, the Pd-based catalysts prepared on activated carbon (Pd/AC) and reduced graphene oxide (Pd/rGO), which give a yield of 36.7% and 48.9%, respectively, manifest markedly inferior performance compared to those immobilized on N-CNS supports (entries 9, 10), highlighting the pivotal role of the support in enhancing the catalytic performance. In comparison, the Pd/N-CNS800 catalyst shows approximately 9-fold higher activity compared to the commercial Pd/C catalyst (entry 11), further accentuating the contribution of high nitrogen-doped carbon nanosheets in improving the catalytic activity.

**Figure 6:**
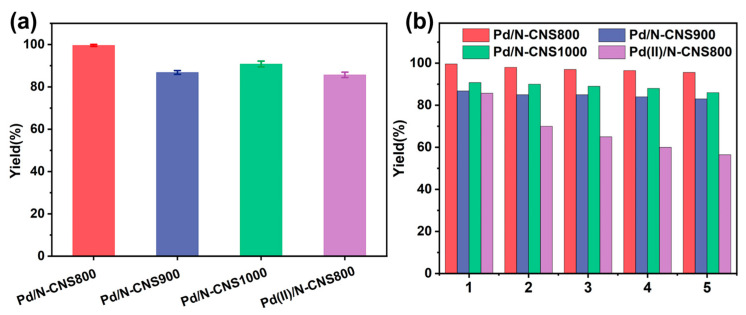
(**a**) The Suzuki-coupling reaction with the Pd/N-CNS and Pd(II)/N-CNS samples. (**b**) The reusability test of the Pd/N-CNS samples and Pd(II)/N-CNS.

To further elucidate the intrinsic factors contributing to the exceptional catalytic performance of the catalysts, a comparative analysis was conducted focusing on the catalytic activity and stability of Pd/N-CNS catalysts versus Pd(II)/N-CNS800. As shown in Figure 6a, the conversion rates for the Pd/N-CNS800, Pd/N-CNS900, and Pd/N-CNS1000 catalysts are recorded at 99.6%, 86.8%, and 90.8%, respectively. Under identical reaction conditions, the yield of Pd(II)/N-CNS800 after 50 min is a mere 85.7%, underscoring the superior activity of supported palladium nanoparticles. After five cycles of reuse, the Pd/N-CNS800 catalyst exhibits no obvious decline in activity, with a maintained overall TOF of 2294 h^−1^, as depicted in Figure 6b. In contrast, the catalytic yield of Pd(II)/N-CNS800 diminishes to 56.5%, indicating its poor stability. Notably, the Pd/N-CNS800 catalyst only shows a minimal decline in activity even after 10 cycles (Figure S6). Subsequent ICP-OES analysis of the recycled catalyst reveals that the Pd content still remains at 0.67 wt% (Table S2), with only a very negligible loss compared to the initial measurement. Based on the comparative analysis of these results, the Pd/N-CNSt catalysts demonstrate excellent catalytic performance in an Ar reaction atmosphere. The enhancement in both activity and stability of the Pd/N-CNS800 catalyst, as compared to the Pd(II)/N-CNS800 catalyst with directly adsorbed Pd²⁺ active sites, is attributed to the high nitrogen doping on the two-dimensional support, which facilitates the transformation of Pd⁰ to Pd²⁺ through electron transfer between the support and the metal, ensuring the secure immobilization of the metal nanoparticles.

**Figure 7:**
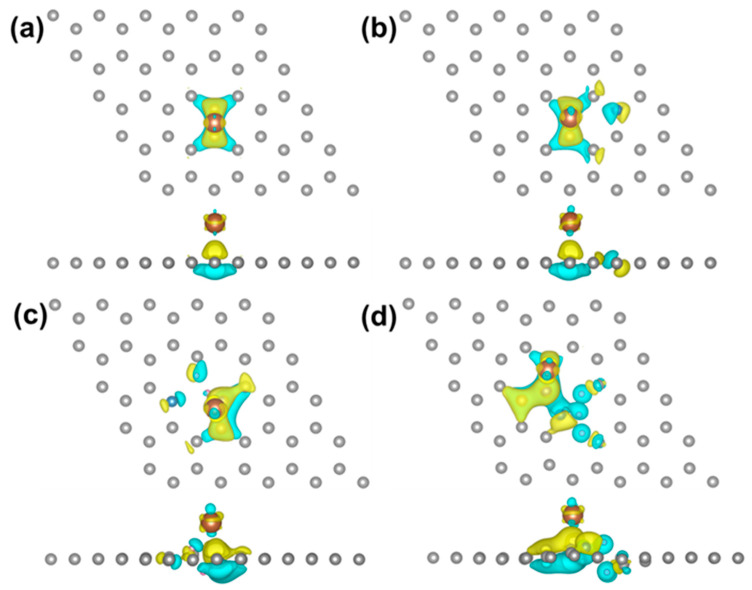
The differential charge density for Pd atom adsorption on models of (**a**) graphene, (**b**) graphitic N, (**c**) pyridinic N, and (**d**) pyrrolic N. The grey, blue, pink, and brown balls stand for carbon, nitrogen, hydrogen, and palladium atoms, respectively. The yellow and blue isosurfaces correspond to the increase in the number of electrons and the depletion zone, respectively.

To further elucidate the beneficial effects of nitrogen doping on the catalytic performance of palladium, density functional theory (DFT) calculations were executed. Four models were conducted and optimized, including graphene structures modified with single atomic nitrogen, graphitic nitrogen, pyridinic nitrogen, and pyrrolic nitrogen dopants, as depicted in Figure S7. In all models, the C-C bridge sites emerge as preferred locations for the adsorption of palladium atoms. Notably, while the graphitic nitrogen model exhibits minimal deformation, the pyridinic and pyrrolic nitrogen models demonstrate significant distortion in the side view, correlating with the surface properties of these two types of defect nitrogen structures. The binding energy (E_b_) and the adsorption height (h) were calculated and are presented in Table S3. The adsorption height (h) is defined as the vertical distance between the palladium atom and the average z-coordinate of the carbon and nitrogen atoms within the graphene layer. The findings reveal no substantial difference in h and E_b_ for palladium atoms adsorbed on graphene and graphitic nitrogen models, indicating a negligible effect of graphitic nitrogen-doped supports on palladium. In contrast, the E_b_ values for the palladium atoms adsorbed on the pyridinic nitrogen and pyrrolic nitrogen models increase to 1.04 and 0.67 eV, respectively, while the height of the palladium atom on the pyridinic nitrogen model decreases to 1.8 Å. Consequently, the incorporation of pyridinic and pyrrolic nitrogen can efficiently enhance metal–support binding, thereby promoting the stability of metal particles. Additionally, Bader charge analysis was performed to calculate the charge transfer number (ΔQ) of the palladium atom (Table S3), and differential charge density maps are presented in Figure 7 to illustrate the increased electron transfer from the palladium atom to the pyridinic nitrogen-doped support. Through a systematic analysis combining experimental characterization and theoretical calculations, it is demonstrated that the surface electronic structure of the two-dimensional carbon nanosheets is significantly improved by the doping of pyridinic nitrogen species. This enhancement facilitates electron transfer from the metal to the support, thereby strengthening the support–metal interaction, which is a crucial factor in reinforcing the catalyst performance.

## 4. Conclusions

In summary, by utilizing petroleum asphalt as a carbon source and g-C_3_N_4_ as an intermediate template, two-dimensional carbon nanosheets with high nitrogen doping were synthesized through an intercalation templating strategy. These nanosheets served as a versatile support for the immobilization of Pd nanoparticles in Suzuki cross-coupling reactions. The Pd/N-CNS catalyst achieved an overall turnover frequency (TOF) of 2390 h^−1^ for the Suzuki cross-coupling reaction under mild conditions, representing approximately a nine-fold increase in activity compared to commercial Pd/C catalysts. Furthermore, this catalyst maintained an overall TOF of 2294 h^−1^ even after five reaction cycles, demonstrating excellent stability. Experimental results and theoretical calculations reveal the significant role of the abundant pyridinic N species in the observed enhancements in catalytic performance, which induces improved electron transfer from Pd to the support, strengthening the support–metal interaction and promoting the catalytic conversion processes. This work provides valuable insights into feasible strategies for developing efficient catalysts aimed at the sustainable production of biaromatic compounds.

## Figures and Tables

**Table 1:** Suzuki-coupling reactions catalyzed by the various catalysts ^a^.

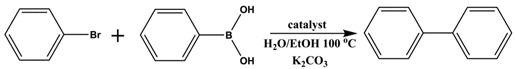				
Entry	Catalyst	Reaction Time (min)	Yield (%)	Final TOF (h^−1^)
1	Blank	50	0	—
2	N-CNS800	50	0.6	—
3	N-CNS900	50	0.5	—
4	N-CNS1000	50	0.8	—
5	Pd/N-CNS800	50	99.6	2390
6	Pd/N-CNS900	50	86.8	2083
7	Pd/N-CNS1000	50	90.8	2179
8	Pd(II)/N-CNS800	50	85.7	2057
9	Pd/AC	50	36.7	881
10	Pd/rGO	50	48.9	1173
11	Commercial Pd/C	50	11.5	276

^a^ Reaction conditions: 1.0 mmol bromobenzene, 1.5 mmol phenylboronic acid, 0.05 mmol% Pd catalyst, 2 mmol K_2_CO_3_, 10.0 mL H_2_O/EtOH (V_H2O_/V_EtOH_ = 1:3), 100 °C, Ar atmosphere.

## Data Availability

Data are contained within the article. The data presented in this study are available.

## Supplementary Materials

The following supporting information can be downloaded at https://www.mdpi.com/article/10.3390/nano14211690/s1: Figure S1: (a) XRD curve and (b) SEM image of BAC; Figure S2: SEM and TEM images of (a,b) N-CNS800, (c,d) N-CNS900, and N-CNS1000 (e,f); Figure S3: (a) XRD and (b) Raman spectra of the N-CNS samples, and (c) FTIR spectra of BAC, N-CNS800, N-CNS900, and N-CNS1000; Figure S4: HR-TEM spectra of the Pd/NCNS samples; Figure S5: Pd 3d high-resolution XPS spectra of (a) Pd_2_(dba)_3_·CHCl_3_ and (b) Pd/AC; Figure S6: The reusability test of the Pd/N-CNS800 catalyst. Figure S7: Top and side views for Pd atom adsorption on models of (a) graphene, (b) graphitic N, (c) pyridinic N, and (d) pyrrolic N. The grey, blue, pink, and brown balls stand for carbon, nitrogen, hydrogen, and palladium atoms, respectively; Table S1: Elemental contents and concentrations in the Pd/N-CNS samples determined by XPS measurements; Table S2: Elemental contents and concentrations in the Pd/N-CNS samples determined by XPS measurements; Table S3: The binding energies (*E*_b_), adatom heights (*h*), and electron transfer of Pd atom (*ΔQ*) for Pd-decorated pristine and N-doped graphene.
