# Supplementary material for: Pd Nanoparticles Immobilized on Pyridinic N-Rich Carbon Nanosheets for Promoting Suzuki Cross-Coupling Reactions
Source: Nanomaterials (Basel). 2024 Oct 22;14(21):1690. doi: 10.3390/nano14211690 (PMC11548024; doi:10.3390/nano14211690)
Supplement: Supplementary file 1 [file nanomaterials-14-01690-s001.zip › nanomaterials-3240780-supplementary.pdf]

Supplementary Information

Pd Nanoparticles Immobilized on Pyridinic N-Rich Carbon Nanosheets for Promoted Suzuki Cross-Coupling Reactions

Shihao Cui [1], Dejian Xu [1], Zhiyuan Wang [1], Libo Wang [1], Yikun Zhao [2], Wei Deng [2], Qingshan Zhao [1,*], and Mingbo Wu [1]

1 State Key Laboratory of Heavy Oil Processing, College of Chemistry and Chemical Engineering, China University of Petroleum (East China), Qingdao 266580, China; cuishihao001124@163.com (S.C.); xudejian666@163.com(D.X.); 18563407980@163.com(Z.W.); wlb1886056@163.com(L.W.) ; wumb@upc.edu.cn

2 Qingdao Chaorui Nanotechnologies Co. Ltd, Qingdao 266600, China; zhaoyikun@chaoruinano.com(Y.Z.); dengwei@chaoruinano.com(W.D.)

*Corresponding author: Qingshan Zhao

E-mail address: qszhao@upc.edu.cn

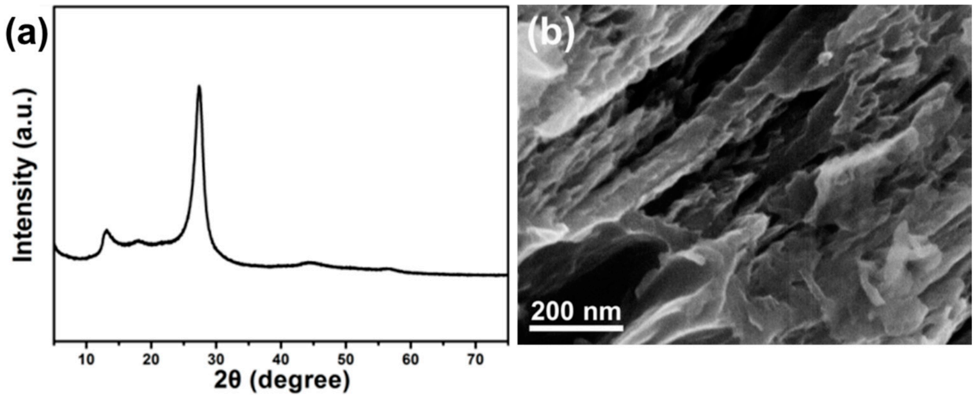

Figure S1. (a) XRD pattern and (b) SEM image of BAC.

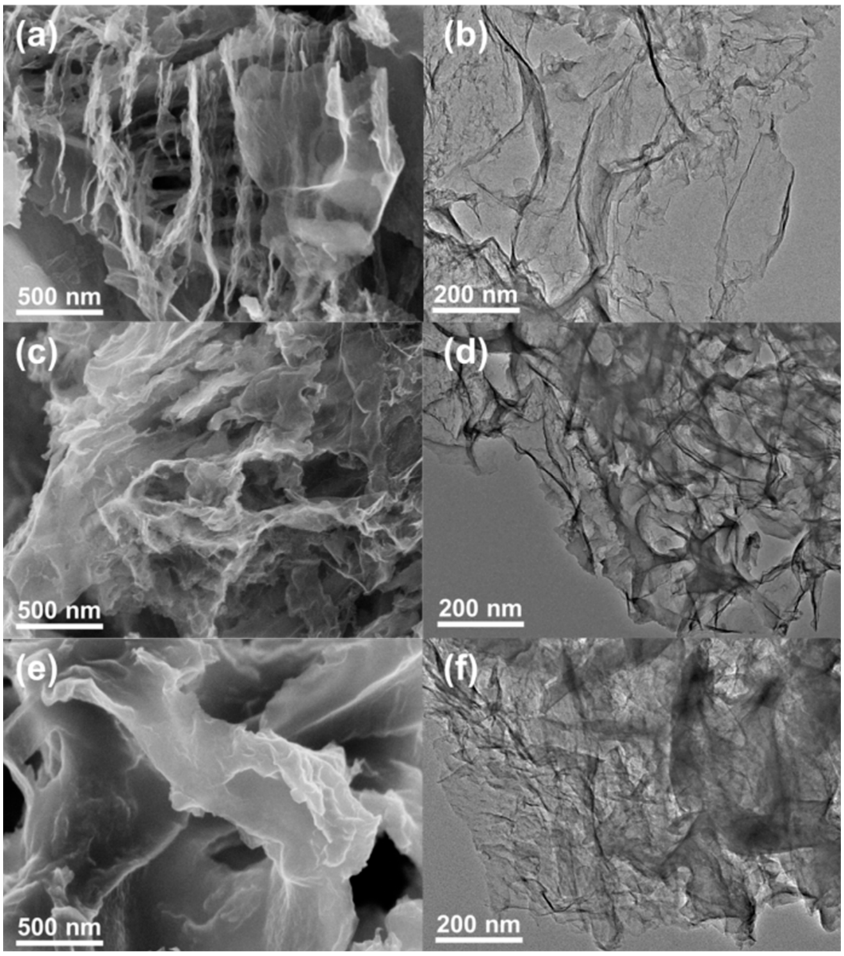

Figure S2. SEM and TEM images of (a-b) N-CNS800, (c-d) N-CNS900, and N-CNS1000 (e-f).

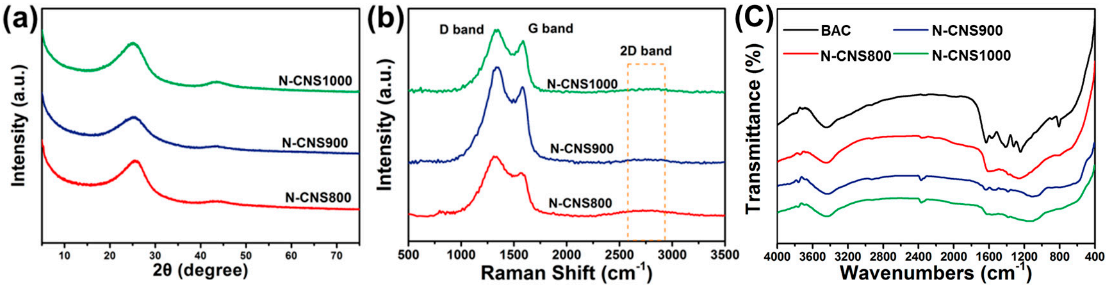

Figure S3. (a) XRD patterns and (b) Raman spectra of N-CNS800, N-CNS900, and N-CNS1000, and (c) FTIR spectra of BAC, N-CNS800, N-CNS900, and N-CNS1000.

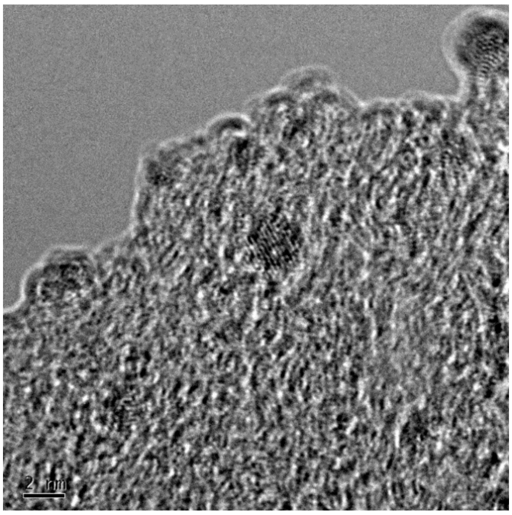

Figure S4. HR-TEM image of the Pd/NCNS800.

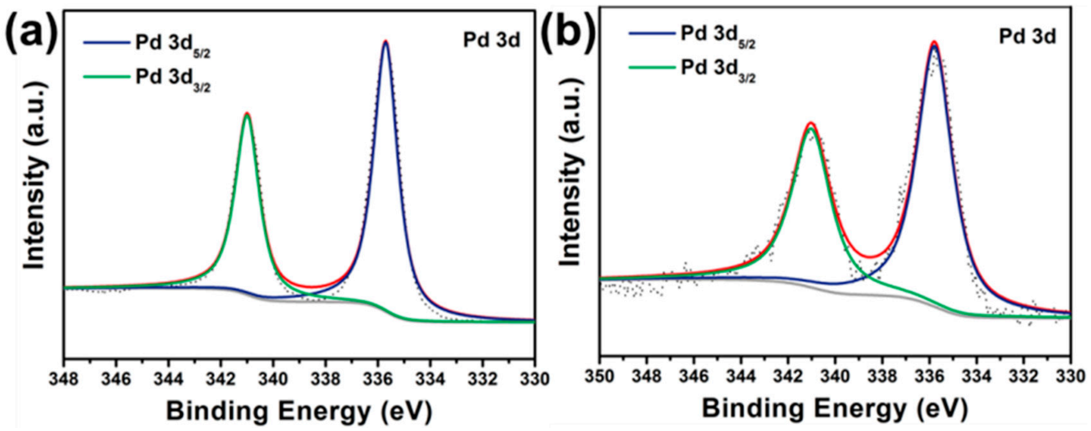

Figure S5. Pd 3d XPS high-resolution spectra of (a) $Pd_2(dba)_3$•$CHCl_3$ and (b) Pd/AC.

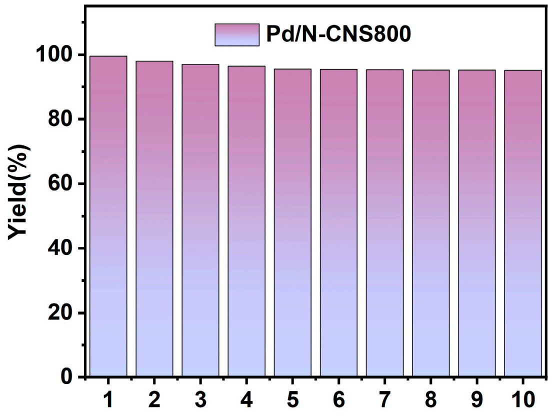

Figure S6. The reusability test of the Pd/N-CNS800 catalyst.

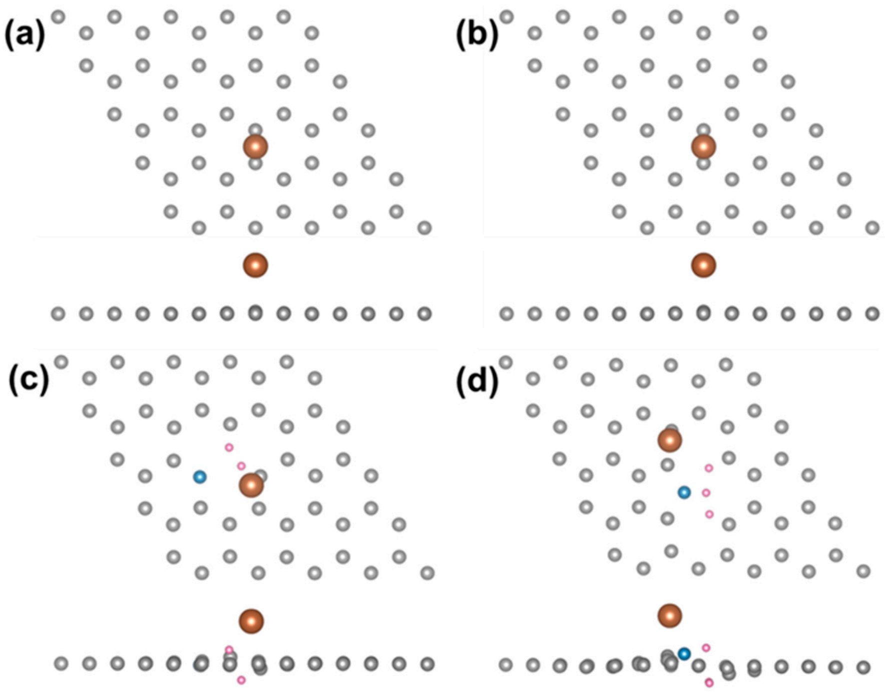

Figure S7. Top and side views for Pd atom adsorption on models of (a) graphene, (b) graphitic N, (c) pyridinic N, and (d) pyrrolic N. The grey, blue, pink, and brown balls stand for carbon, nitrogen, hydrogen, and palladium atoms, respectively.

Table S1. Elemental contents and concentrations in the Pd/N-CNS samples determined by XPS measurements

Sample	Surf.(m^2g^{-1})	Micro. Surf.(m^2g^{-1})	Micro. Volume(cm^3g^{-1})	I_D/I_G
N-CNS800	286.8	144.6	0.075	1.16
N-CNS900	124.3	12.6	0.005	1.11
N-CNS1000	121.2	2.5	0.0001	1.05

Table S2. Elemental contents and concentrations in the Pd/N-CNS samples determined by XPS measurements.

Sample	C	N	O	Pd			Pyridinic N	Pyrrolic N	Graphitic N	Oxidized N	Pd^0	Pd^{2+}
	(at.%)	(at.%)	(at.%)	(at.%)	(wt.%)[a]	(wt.%)[b]	(at.%)	(at.%)	(at.%)	(at.%)	(at.%)	(at.%)
Pd/N-CNS800	80.19	14.18	5.00	0.63	0.69	0.67	6.59	4.49	2.39	0.71	11.0	89.0
Pd/N-CNS900	85.55	8.06	5.75	0.64	1.03	0.99	2.45	2.13	2.58	0.90	53.2	46.8
Pd/N-CNS1000	88.65	5.96	4.79	0.59	1.24	1.20	1.81	0.49	2.65	1.01	60.1	39.9

[a] Measured by ICP-OES.

[b] Measured by ICP-OES of the recycled catalyst.

Table S3. The binding energies (E_b), adatom heights (h), electron transfer of Pd atom (ΔQ) for Pd-decorated pristine and N-doped graphene.

System	Graphene	Graphitic	Pyridinic	Pyrrolic
h (Å)	2.1	2.1	1.8	2.2
E_b (eV)	0.49	0.49	1.04	0.67
ΔQ (e)	-0.27	-0.27	-0.31	-0.26
